# High prevalence of type 2 diabetes among the urban middle class in Bangladesh

**DOI:** 10.1186/1471-2458-13-1032

**Published:** 2013-10-31

**Authors:** Nazmus Saquib, Masuma Akter Khanam, Juliann Saquib, Shuchi Anand, Glenn M Chertow, Michele Barry, Tahmeed Ahmed, Mark R Cullen

**Affiliations:** 1 Stanford University School of Medicine, Stanford Prevention Research Center (SPRC), Stanford University, Stanford, USA; 2 Centre for Control of Chronic Diseases, International Center on Diarrheal Diseases and Research (ICDDR, B), Dhaka, Bangladesh; 3 Centre for Clinical Epidemiology and Biostatistics, University of Newcastle, Newcastle upon Tyne, Australia; 4 Stanford University School of Medicine, Division of Nephrology, Stanford University, Stanford, USA; 5 Stanford University School of Medicine, Global Health, Stanford University, Stanford, USA; 6 Centre for Nutrition and Food Security, International Center on Diarrheal Diseases and Research (ICDDR, B), Dhaka, Bangladesh; 7 Stanford University School of Medicine, General Medical Disciplines, Stanford University, Stanford, CA, USA

## Abstract

**Background:**

The prevalence of type-2 diabetes and metabolic syndrome are increasing in the developing world; we assessed their prevalence among the urban middle class in Bangladesh.

**Methods:**

In this cross-sectional survey (n = 402), we randomly selected consenting adults (≥ 30 years) from a middle-income neighborhood in Dhaka. We assessed demography, lifestyle, and health status, measured physical indices and blood pressure and obtained blood samples. We evaluated two primary outcomes: (1) type-2 diabetes (fasting blood glucose ≥ 7.0 mmol/L or hemoglobin A1C ≥ 6.5% (48 mmol/mol) or diabetes medication use) and (2) insulin resistance (type-2 diabetes or metabolic syndrome using International Diabetes Federation criteria).

**Results:**

Mean age and Quételet’s (body mass) index were 49.4 ± 12.6 years and 27.0 ± 5.1 kg/m^2^; 83% were married, 41% had ≥12 years of education, 47% were employed, 47% had a family history of diabetes. Thirty-five percent had type-2 diabetes and 45% had metabolic syndrome. In multivariate models older age and family history of diabetes were significantly associated with type-2 diabetes. Older age, female sex, overweight or obese, high wealth index and positive family history of diabetes were significantly associated with insulin resistance. Participants with type-2 diabetes or insulin resistance had significantly poorer physical health only if they had associated cardiovascular disease.

**Conclusions:**

The prevalence of type-2 diabetes and metabolic syndrome among the middle class in Dhaka is alarmingly high. Screening services should be implemented while researchers focus on strategies to lessen the incidence and morbidity associated with these conditions.

## Background

Chronic disease prevalence has increased dramatically in Bangladesh in the last two decades, as has associated mortality [1,2]. Chronic, non-communicable diseases accounted for 8% of total mortality in 1986 and 68% of total mortality in 2006 [2]. It has been proposed that the burden of disease can be explained, in part, by the higher prevalence of adverse lifestyle-related risk factors in the population, and may therefore be preventable [3]. For instance, centripetal obesity is common among Bangladeshis, particularly among women, in whom the prevalence has been reported as high as 60% to 75% [4,5]. Consequently, Bangladeshis are at increased risk for developing metabolic syndrome and type-2 diabetes. Current estimates suggest that prevalence of type-2 diabetes is between 2% and 21% [1] and prevalence of metabolic syndrome between 3% and 20% [6-8].

The available prevalence estimates of type-2 diabetes and metabolic syndrome have been derived from either rural or urban samples that include slum-dwellers [1]. Bangladesh now has an emerging middle class which has not been carefully evaluated. There are reasons to believe that the prevalence of type-2 diabetes and metabolic syndrome might be elevated in the middle class. Sustained economic growth has enabled the new middle class to consume higher intakes of food and to choose higher-calorie and so-called “fast-food” options more frequently [9]. Moreover, the city neighborhoods are not conducive to safe outdoor activities due to the confluence of population density, traffic jams, and crime; other prohibitive factors include a hot and humid climate, unremitting construction work, and excessive dust [10,11].

This study evaluated the prevalence and correlates of type-2 diabetes and metabolic syndrome in a sample of middle-class men and women living in the capital Dhaka. We used a rigorous method for sample selection, practical criteria for assessing socio-economic status of the participants, and objective measures to define disease prevalence. We hypothesized that type-2 diabetes and metabolic syndrome prevalence would be higher among the middle class compared to estimates previously published from urban-based samples in Bangladesh.

## Methods

### Sample

We conducted this cross-sectional study among 402 adult (≥30 years) men and non-pregnant women who were residents of a middle-income neighborhood in Dhaka, Bangladesh. The institutional review board at Stanford University School of Medicine and the ethical review committee at International Center for Diarrheal Diseases, Bangladesh (ICDDR,B) approved the study protocol.

### Sampling strategy

We selected participants through a multi-stage random sample procedure. We randomly selected three out of six blocks of Mohammedpur -- the middle-income neighborhood identified for this study -- using a detailed area map. We gave all residential buildings of the selected blocks a unique identification number to generate a list; from that list we randomly selected 500 buildings. Selection of extra buildings (25%) ensured an *a priori* restriction of enrollment of one person per building with a sample size goal of 400. Most of residential buildings in Mohammedpur are apartment complexes, the remaining are single family houses. If the building was an apartment complex, we counted the total number of households, and consulted a random number table to select a single household. We determined the eligible number of participants in the selected household. If there were only one, then we recruited this person; however, if there were more than one, then we randomly selected a single occupant. If there was no eligible person in a selected household or the eligible person declined to participate, we randomly chose another household within the same apartment complex. We enrolled participants according to a pre-specified quota (male and female; 30–50 and >50 years) in order to ensure adequate sex and age representation (i.e., approximately 100 from each combination). We enrolled participants until the quota for any given combination was reached, after which participants were enrolled only into the remaining strata.

### Data collection

Research assistants described the study purpose and procedures, which included two visits to participants’ homes -- one for interview and another for biological sample collection. Participants gave their informed consent and were enrolled into the study. Research assistants conducted face-to-face interviews for approximately one hour with standardized questionnaires and obtained information on demography, socioeconomic status, lifestyle factors such as diet, physical activity, and tobacco use, self-reported personal and family history of chronic diseases, and health-related quality of life. At the end of interview, research assistants consulted the participants to set a suitable date and time (usually within a week of the first interview) for a second visit in the early morning, and instructed the participants to fast for 12 hours during the night before their second visit.

On the second visit, the study nurse measured the participant’s height, weight, waist and hip circumferences according to standard protocols and recorded two blood pressure readings with a sphygmomanometer after the participant had been in a seated position for five minutes with his or her arm resting on the chair arm. The nurse also collected a fasting blood sample (5–10 mL) from the antecubital space with a vaccutainer. A total of 358 out of 402 participants provided blood samples.

### Laboratory analysis

We immediately transported the blood samples to the ICCDR,B laboratory, where samples were centrifuged to obtain serum. A biochemical analysis of serum was done for fasting blood glucose, hemoglobin A1c (HbA1c), triglycerides, total cholesterol, and HDL cholesterol (all measured in mmol/L) [reagent and analyzer model: Beckman Coulter, AU680 & 640]. Assay-variation was checked daily with an internal quality assessment scheme and the coefficient of variation for each test was maintained below 5%. Participants with abnormal test results received their medical report and were referred to a local physician.

### Study outcome

**Figure 1:**
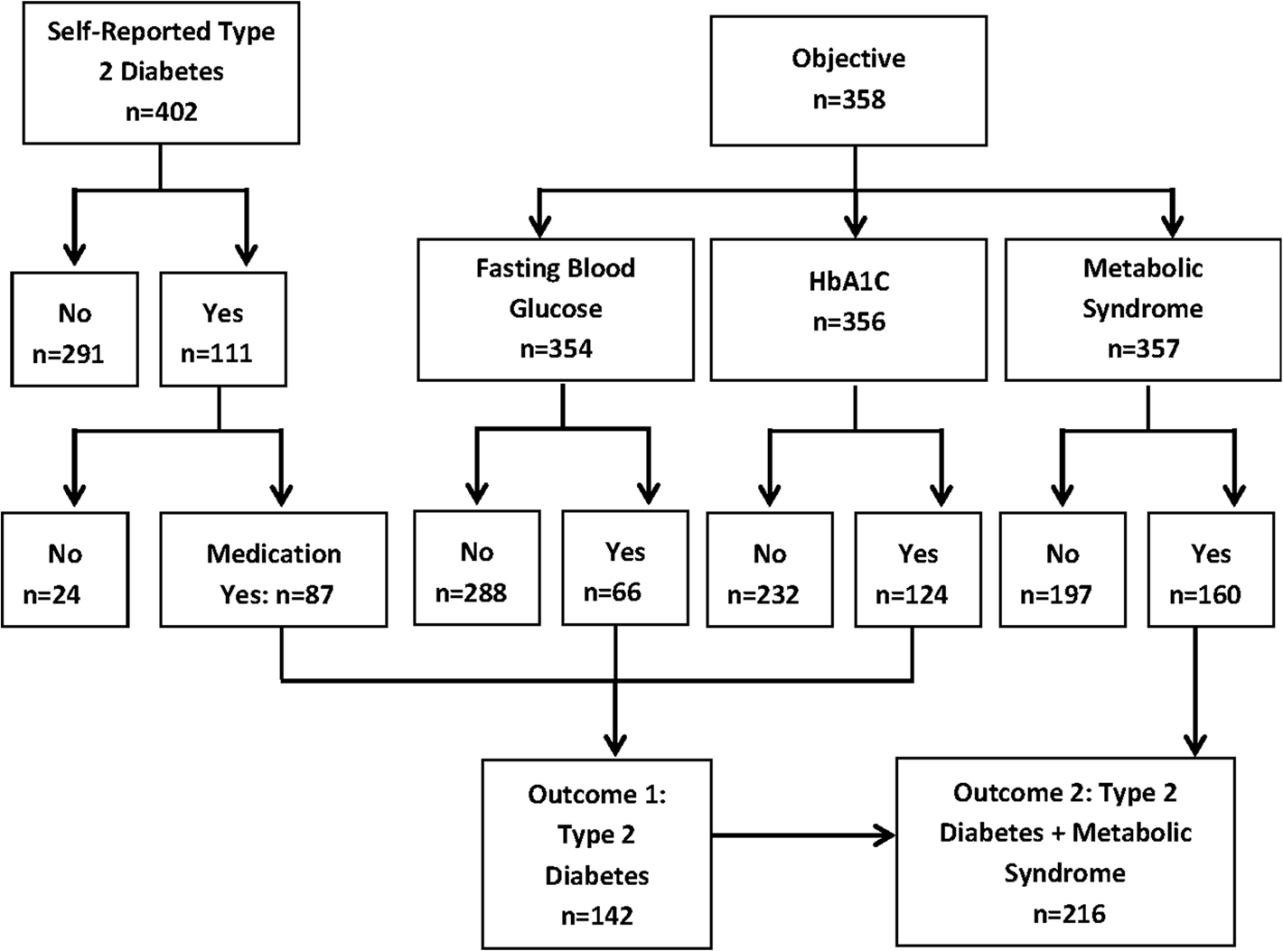
**Flowchart of outcomes (1) Type-2 diabetes and (2) Insulin Resistance (type-2 diabetes or Metabolic Syndrome).** Type-2 diabetes: elevated fasting glucose or hemoglobin AIC or medication use for self-reported diabetes. Metabolic Syndrome: enlarged waist circumference (male: ≥ 90 cm, female: ≥ 80 cm) plus any 2 of the following 4 risk factors: elevated blood pressure, fasting plasma glucose, or triglycerides, or a decreased high-density lipoprotein.

We defined type-2 diabetes as fasting blood glucose ≥ 7.0 mmol/L (or 126 mg/dl) or HbA1c ≥6.5% (48 mmol/mol) or medication use for self-reported diabetes (12). We defined metabolic syndrome according to International Diabetes Federation criteria [12] as centripetal obesity (waist circumference ≥90 cm for men and ≥80 cm for women) plus any two of the followings: (1) triglycerides >150 mg/dL (1.7 mmol/L) or treatment for elevated triglycerides, (2) HDL cholesterol <40 mg/dL (1.03 mmol/L) in men or <50 mg/dL (1.29 mmol/L) in women, or treatment for low HDL (3) systolic blood pressure >130 mmHg, diastolic blood pressure >85 mmHg, or treatment for hypertension, and (4) fasting plasma glucose >100 mg/dL (5.6 mmol/L) or previously diagnosed diabetes mellitus. Two outcomes were evaluated in the analysis: (1) type-2 diabetes and (2) insulin resistance (type-2 diabetes or metabolic syndrome (Figure 1)).

### Covariates

We considered the following demographic and life-style variables: age (30–40, 41–50, 51–60, >60 years), sex (Male, Female), education (<5 years, 5–12 years, and >12 years), currently married (yes, no) or employed (yes, no), smoking status (never, former, and current), current use of smokeless tobacco (yes, no), living in Dhaka (native, migrant). We categorized participants based on their calculated Quetelet’s body mass index (BMI, in kg/m^2^) as lean or normal (< 25), overweight (25–29.9), and obese (≥ 30) using standard as opposed to race-specific cut-off values. We evaluated socioeconomic status with an objective wealth index that used four items: whether the participant owned an apartment or house in the city, a car, an air-conditioner, or an instant power generator (as power outage is common in Dhaka) at the residence. Participants received one point for possessing each item; a participant could tally as high as four and as low as zero points.

Participants reported chronic conditions diagnosed by a physician. We considered a participant to have cardiovascular disease (CVD) if he or she reported medication use for either hypertension or heart disease or if his or her blood pressure was ≥140/90 mmHg during measurement.

We assessed health-related quality of life with the Medical Outcomes Study Short form 12 (SF-12), which includes four physical (i.e., general health, physical functioning, bodily pain, and role limitations due to physical health) and four mental (i.e., mental health, vitality, role limitations due to emotional problems, and social functioning) subscales [13]. For each subscale, we calculated overall scores by summing responses to the individual questions linked to that particular subscale. The physical and the mental component summary scores (PCS and MCS, respectively) included all eight subscales in the computation. A higher PCS (or MCS) score indicated better physical (or mental) health. These scales have been previously used in the Bangladeshi population and were shown to be valid and reliable [15].

### Statistical analysis

We checked variables for accuracy and generated sex-specific descriptive statistics for demography, lifestyle, chronic conditions, and socio-economic status. We ran frequencies of metabolic syndrome criteria (centripetal obesity, elevated triglycerides and low high-density lipoprotein, hypertension, and hyperglycemia). We calculated and graphed the strata-specific (age, sex, BMI, and wealth index) prevalence of study outcomes.

We employed logistic regression to assess the correlates of study outcomes. The unadjusted models included each outcome with the following covariates: age, sex, BMI, education, marital and employment status, smoking status, smokeless tobacco use, physical activity, duration of living in the city, family history of diabetes, and wealth index. We considered all covariates for the inclusion in the adjusted outcome models and applied a backward selection procedure, in which non-significant variables were deleted one by one until only variables significant at p < 0.05 were included.

We assessed physical and mental component summary scores according to the presence or absence of study outcomes. Since CVD is common among persons with type-2 diabetes and is associated with health-related quality of life, we examined self-reported physical and mental health according to the cross-strata of each outcome (type-2 diabetes and insulin resistance) and CVD. For an example, participants could be in one of the four groups when type-2 diabetes was the outcome: (1) no type-2 diabetes or cardiovascular disease (reference group), (2) type-2 diabetes only, (3) cardiovascular disease only, and (4) both type-2 diabetes and cardiovascular disease. We employed generalized linear models to obtain participants’ mean physical and mental composite score by group after adjusting for age; we contrasted the scores between the reference and the comparison groups using a Bonferroni correction for multiple comparisons. We analyzed the data using SAS version 9.2 (Cary, NC), and considered two-sided inference tests with an alpha <0.05 as statistically significant.

## Results

### Demographic and lifestyle factors

The sample had comparable distribution by age strata and sex as shown in Table 1. Three-fourths of the participants had migrated to Dhaka. The proportion with university-level education or employment was higher among men. Although 31% of participants reported cigarette smoking (former and current), smoking was rare among women (<3%). Conversely, the prevalence of smokeless tobacco use was higher among women. Nearly two-thirds of participants were either overweight or obese; the prevalence was much higher among women.

**Table 1:** Demographic characteristics of the urban Bangladeshi sample according to sex (n = 402)

**Variable**	**Men n = 209**	**Women n = 193**	**Overall n = 402**
	**Percent or mean (SD)**	**Percent or mean (SD)**	**Percent or mean (SD)**
Mean age	49.7 (12.3)	49.1 (13.1)	49.4 (12.7)
Living in Dhaka (%)			
Native	21.5	35.7	28.3
Migrant	78.5	64.3	71.6
Education (years)			
< 5: primary	9.1	22.8	15.6
6 to 12: school + some college	37.8	48.7	43.0
>12: university	53.1	28.5	41.3
Marital status (% married)	93.3	72.5	83.3
Employment (% employed)	75.1	16.6	47.0
Quételet’s index (kg/m^2^)			
Lean or normal, < 25.0 (%)	49.3	23.3	36.8
Overweight, 25.1 -30.0 (%)	38.3	46.1	42.0
Obese, >30.0 (%)	12.4	30.6	21.1
Smoking			
Non-smoker	42.1	97.4	68.6
Former smoker	23.9	2.6	13.6
Current smoker	34.0	0.0	17.6
Smoke-less tobacco (% current users)	10.1	21.2	15.4
Family history of diabetes (%)	45.4	49.2	47.3
Wealth index*			
0	37.3	29.5	33.6
1-2	48.3	54.9	51.5
3-4	14.3	15.5	14.9

* based on owning an apartment/house, private car, instant power source, or air conditioner; one point for each item.

### Type-2 diabetes mellitus and metabolic syndrome

**Figure 2:**
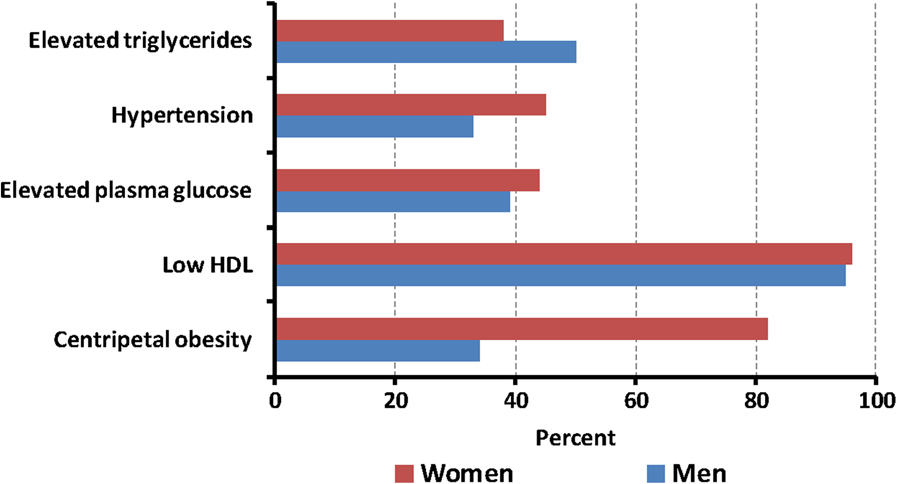
**Prevalence of individual components of Metabolic Syndrome by sex.** Components included: centripetal obesity: (waist circumference ≥90 cm for men and ≥80 cm for women) plus any two of the following: (1) elevated triglycerides: triglycerides >150 mg/dL (1.7 mmol/L), (2) Low HDL: high-density lipoprotein <40 mg/dL (1.03 mmol/L) in men or <50 mg/dL (1.29 mmol/L) in women, (3) Hypertension: systolic blood pressure >130 mmHg, diastolic blood pressure >85 mmHg, or treatment for hypertension, and (4) Elevated plasma glucose: fasting plasma glucose >100 mg/dL (5.6 mmol/L) or previously diagnosed diabetes mellitus.

**Figure 3:**
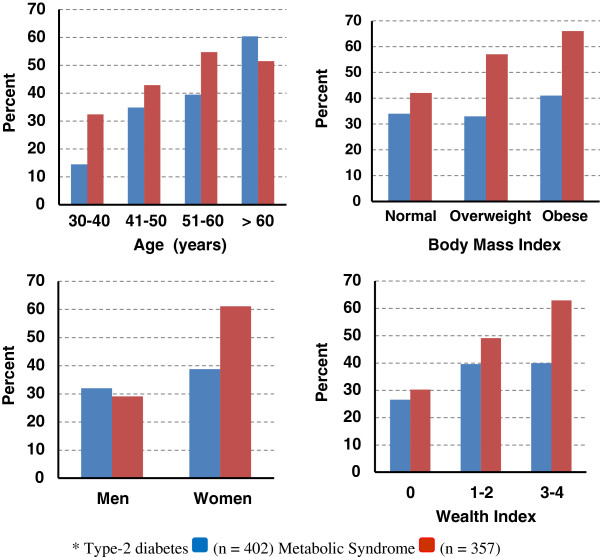
**Prevalence of Type-2 diabetes (n = 402) and Metabolic Syndrome (n = 357) by age, body mass index, sex, and wealth index.** Wealth index is based on owning an apartment/house, private car, instant power source, or air conditioner; one point for each item.

The prevalence of centripetal obesity, elevated triglycerides, and elevated blood pressure was higher among women than men whereas the prevalence of low HDL cholesterol and high plasma glucose was comparable between the sexes (Figure 2). A total of 35% (142/402) of participants had type-2 diabetes (elevated plasma glucose = 19%, elevated HbA1c = 35%, both elevated plasma glucose and elevated HbA1c = 18%, medication use for type-2 diabetes = 22%); and 45% (160/357) had metabolic syndrome. Further, 15% (52/357) had type-2 diabetes without metabolic syndrome, 21% (74/357) had metabolic syndrome without type-2 diabetes and 24% (86/357) had both conditions. The prevalence of type-2 diabetes and metabolic syndrome was generally higher among participants who were female, older, overweight/obese, and scored higher in the wealth index (Figure 3). More than half of all participants (54%) had insulin resistance.

### Unadjusted and adjusted associations with study outcomes

Table 2 shows the results of the regression models. Participants who were older, currently not married or not employed, had a family history of diabetes, and had a higher wealth index (1–2 or 3–4) were more likely to have type-2 diabetes and insulin resistance in the unadjusted models. In addition, smokeless tobacco users were more likely to have type-2 diabetes; and women and those who were overweight or obese were more likely to have insulin resistance. Neither urbanization nor education was strongly associated with either outcome.

**Table 2:** Unadjusted and adjusted associations with disease outcomes: (1) type-2 diabetes and (2) insulin resistance (type-2 diabetes or metabolic syndrome); n = 402

**Variable**	**Type-2 diabetes**		**Insulin resistance**	
	**Unadjusted**	**Adjusted**	**Unadjusted**	**Adjusted**
	**OR (95% CI)**	**OR (95% CI)**	**OR (95% CI)**	**OR (95% CI)**
Age				
30 to 40 (ref)	1.0	1.0	1.0	1.0
41 to 50	3.17 (1.64, 6.14) *	2.97 (1.51, 5.86) *	1.95 (1.13, 3.36) *	1.92 (1.08, 3.44) *
51 to 60	3.86 (1.99, 7.47) *	3.73 (1.89, 7.34) *	3.07 (1.76, 5.36) *	3.44 (1.88, 6.30) *
>60	9.00 (4.48, 18.1) *	7.02 (3.31, 14.9) *	5.96 ( 3.11, 11.4) *	7.16 (3.51, 14.6) *
Sex				
Male (ref)	1.0		1.0	1.0
Female	1.34 (0.89, 2.03)		2.01 (1.35, 3.00) *	1.84 (1.17, 2.89) *
Education (years)				
University (ref)	1.0		1.0	
High school + college	1.16 (0.73, 1.82)		0.98 (0.64, 1.50)	
Primary school	1.70 (0.94, 3.09)		1.47 (0.81, 2.66)	
Currently married				
Yes (ref)	1.0	1.0	1.0	1.0
No	2.33 (1.37, 3.96) *	1.69 (0.92, 3.09)	2.12 (1.21, 3.71) *	1.62 (0.84, 3.14)
Currently employed				
Yes (ref)	1.0	1.0	1.0	
No	2.21 (1.45, 3.38) *	1.43 (0.88, 2.32)	2.51 (1.68, 3.75) *	
Living in Dhaka				
Native (ref)	1.0		1.0	
Migrant	1.13 (0.71, 1.78)		0.96 (0.62, 1.49)	
Body mass index (kg/m^2^)				
Lean or normal, <25.0 (ref)	1.0		1.0	1.0
Overweight, 25.1 -30.0	0.94 (0.59, 1.50)		1.81 (1.16, 2.84) *	1.88 (1.12, 3.13) *
Obese, >30.0	1.33 (0.76, 2.30)		2.60 (1.49, 4.53) *	2.17 (1.15, 4.09) *
Smoking				
Non-smoker (ref)	1.0		1.0	
Former smoker	1.28 (0.71, 2.31)		0.87 (0.48, 1.55)	
Current smoker	0.70 (0.39, 1.24)		0.64 (0.37, 1.08)	
Smoke-less tobacco use				
No (ref)	1.0		1.0	
Yes	2.06 (1.19, 3.56) *		1.44 (0.82, 2.50)	
Family history of diabetes				
No (ref)	1.0	1.0	1.0	1.0
Yes	2.01 (1.32, 3.04) *	2.17 (1.38, 3.41) *	2.07 (1.38, 3.09) *	1.98 (1.26, 3.10) *
Wealth index Ψ				
0 (ref)	1.0		1.0	1.0
1-2	1.80 (1.12, 2.89) *		1.83 (1.18, 2.84) *	1.14 (0.70, 1.88)
3-4	1.83 (0.96, 3.48)		3.56 (1.84, 6.88) *	1.84 (0.89, 3.77)

Logistic regression was used to build the adjusted model.

* indicates p-value < 0.05.

For each outcome the most parsimonious model was selected and odds ratios (95% CI) are provided for the variables that remained in the final model. Any variable without an estimate indicates that it was not retained in the final model.

Ψ based on owning an apartment/house, private car, instant power source, or air conditioner; one point for each item.

In the adjusted model, age and family history of diabetes were significantly associated with type-2 diabetes; whereas age, sex, BMI and family history of diabetes were significantly associated with insulin resistance.

### Health-related quality of life

Self-reported physical and mental health did not vary according to presence of type-2 diabetes or insulin resistance. However, participants with type-2 diabetes or insulin resistance, in conjunction with cardiovascular disease, had a significantly lower PCS score after adjusting for age and multiple comparisons. Participants’ mental component summary score did not differ by disease status (Table 3).

**Table 3:** Health-related quality of life by health conditions (n = 402)

			**SF-12 quality of life**	
			**Physical component summary**	**Mental component summary**
	**Category**	**n**	**Mean ± SE**	**Mean ± SE**
Type-2 diabetes	No diabetes (ref)	260	39.9 ± 0.57	41.2 ± 0.72
	Diabetes	142	38.9 ± 0.79	40.9 ± 0.98
	No diabetes or cardiovascular disease (ref)	168	40.6 ± 0.75	41.5 ± 0.94
	Diabetes only	63	41.1 ± 1.13	42.6 ± 1.42
	Cardiovascular disease only	92	39.0 ± 0.95	40.9 ± 1.19
	Diabetes + cardiovascular disease	79	36.7 ± 1.07*	39.4 ± 1.35
Insulin resistance	No insulin resistance (ref)	186	40.3 ± 0.67	41.1 ± 0.84
	Insulin resistance	216	38.9 ± 0.62	40.9 ± 0.78
	No insulin resistance or cardiovascular disease (ref)	142	40.1 ± 0.79	42.0 ± 1.00
	Insulin resistance only	89	41.6 ± 0.94	41.5 ± 1.19
	Cardiovascular disease only	44	41.4 ± 1.35	39.5 ± 1.72
	Insulin resistance + cardiovascular disease	127	36.8 ± 0.83*	40.4 ± 1.05

p = value * < 0.05.

Means are adjusted for age and for multiple comparisons (Bonferroni).

## Discussion

Our hypothesis was that the diabetes prevalence would be high in the Bangladeshi urban middle class based on our finding of a secular trend in diabetes prevalence in the general population between 1995 and 2010 [1]. In that review, we showed that prevalence was much higher in the urban areas (8.1%) than in the rural areas (4.0%) [1]. There was no reliable estimate for the middle class as the few studies that used an urban sample included slum-dwellers and urban-poor, and analyses stratified by socio-economic status were not presented [4,14]. For example, 21% participants had type-2 diabetes in an urban study that surveyed three neighborhoods in Dhaka in 2008, but it was not clear how socioeconomic status was assessed or what percentage of its participants were truly middle class [14]. While we anticipated a relatively high prevalence in the middle class Bangladeshi population, the actual prevalence far exceeded our expectations.

In this sample, the prevalence of metabolic syndrome was also high. Published data about metabolic syndrome have been derived principally from rural samples; data show that the prevalence of metabolic syndrome has dramatically increased in rural areas. The prevalence in the rural area was only 3% in 2001 [6] and had increased to 20% in 2012 [7]. A recent study on Bangladeshi male immigrants to the United States, which may reflect a population of higher socioeconomic class, reported a prevalence of 38% [15].

Development of type-2 diabetes and metabolic syndrome at a young age, lack of awareness regarding diabetes status, and presence of cardiovascular disease among the majority of persons with type-2 diabetes are additional key findings of this study. In the 30–40 years age bracket approximately 15% and 31% participants had type-2 diabetes and metabolic syndrome respectively. Both these figures are in line with a report that Bangladeshis develop type-2 diabetes at a younger age now than in the past [16]. Nearly 17% of participants who thought they did not have type-2 diabetes were diagnosed with it, and half of the newly diagnosed cases were younger than 50. These findings stress the need to encourage the more affluent section of Bangladeshi society to seek annual health check-ups at an earlier age (30 instead of 40 years), particularly if they are overweight, smoking, or have a family history of type-2 diabetes. Finally, cardiovascular disease was evident in more than half of cases diagnosed with type-2 diabetes and metabolic syndrome (56% and 59%, respectively). These participants scored relatively poorer in the physical component summary score. A poor physical health score has been shown as a strong, independent predictor of mortality in adjusted models for diseased and general populations [17,18].

There are several possible reasons for the high prevalence of diabetes and metabolic syndrome amount urban middle class Bangladeshis. Lifestyle-related risk factors of chronic diseases have a high presence in South Asia [3] and Bangladeshis have the worst profile among the nations in the region. For example, 43% of Bangladeshi participants in the INTERHEART study exhibited centripetal obesity [19]. In our Dhaka sample, 58% have centripetal obesity and 63% were overweight or obese according to BMI; the prevalence was particularly high among women (82% and 77%, respectively). Also, 58% of men had a cigarette-smoking history and 34% were currently smoking. Cigarette smoking is uncommon among Bangladeshi women; however, 21% used tobacco with betel-leaf, a common practice among Bangladeshis.

The high prevalence of overweight and obesity may not fully explain the problem. Changing diet composition as well as excess energy intake may also be contributing factors. The middle class are increasingly exposed to processed and other salt- and chemical-rich food [9]. Nutritional information labeling on packaged food is not enforced and food adulteration is rampant due to poor regulation and limited oversight [20,21]. Further, a lack of nutritional knowledge, cultural preferences and beliefs can inhibit informed decision making about dietary intake and other lifestyle factors.

The physical environment of Dhaka is also likely a contributing factor. Sidewalks, when present, are often narrow and broken, or occupied in this over-populated, largely-unplanned city. Constant traffic jams make it very hard to move from one place to another. The seasonal attributes (hot and humid in summer, dusty in winter, and monsoons during rainy season) make outdoor physical activity both unsafe and unattractive [10].

An important finding of this study is that sex was associated with insulin resistance but not with diabetes. This can be explained by the significant sex difference in central obesity, which is one of the criteria of metabolic syndrome definition and therefore a component of insulin resistance. Since the large sex difference in central obesity did not impact the diabetes prevalence, it raises the question of whether the cutoff point for obesity among women needs to be revised. Future studies should examine the association between central obesity and diabetes among women.

The study strengths include rigorous sampling methods, objective outcome measures, and innovative assessment of participants’ socioeconomic conditions. We maintained random selection in every stage of the sampling frame. We used physical measurement and biological data to define the conditions; hence, the accuracy of disease estimates should be high. Rather than asking participants’ about their income, which participants are either reluctant to provide or often provide with inaccuracy, we selected region-specific possessions to capture socioeconomic status. These possessions are symbols of status and provide comfort to daily living, which can only be purchased by those with a certain degree of financial capacity in a developing country like Bangladesh. The fact that 70% of the participants possessed at least one item is indicative that we have a sample representative of the middle class.

Beyond this, the study has important limitations. We used self-reported data on type-2 diabetes to calculate prevalence for 44 participants who did not provide blood samples. The agreement of type-2 diabetes among self-reported and laboratory data elements was high (83%) for the other 358 participants among whom we have both. Refusal to participate in our survey was 32% (186/588); and those who declined tended to be younger and were more likely to be male and employed. However, the self-reported disease data (e.g. heart disease, type-2 diabetes, and HTN) were comparable between those who participated and those who refused.

## Conclusions

Findings of this study raise a number of research questions that we intend to pursue in future work: (1) identification of the root causes of this sharp rise in type-2 diabetes among the middle class; (2) the incidence (rather than prevalence) of type-2 diabetes and associated prognosis and (3) whether the prevalence of type-2 diabetes, among the middle class, differs between Dhaka and the other urban areas of the country. Answers to these questions are essential to assess current public health policies and clinical practices and to inform changes in policy and practice in light of a diabetes epidemic. In the meantime, resources should be directed at increasing diabetes awareness and education with an emphasis on encouraging health behaviors that are protective and discouraging behaviors that put people at risk. In addition to education on the importance of routine health check-ups for early detection of type-2 diabetes and metabolic syndrome, screening programs should be implemented.

## Competing interests

The authors have no competing interests to declare. This work was supported by the National Institute of Diabetes and Digestive and Kidney Diseases at the National Institutes of Health [K24 DK085446] an internal institutional grant from Stanford University General Medical Disciplines.

## Authors’ contributions

All authors were involved in the design of the study including sampling frame, endpoint selection and data collection and analysis plan. MAK and TA assisted with the data collection plan and supervised field data collection. NS and JS conducted the data analysis and drafted the initial manuscript. All authors reviewed, edited and approved the final the manuscript.

## Pre-publication history

The pre-publication history for this paper can be accessed here:

http://www.biomedcentral.com/1471-2458/13/1032/prepub
